# Gene polymorphism analysis of *Yersinia enterocolitica* outer membrane protein A and putative outer membrane protein A family protein

**DOI:** 10.1186/1471-2164-15-201

**Published:** 2014-03-16

**Authors:** Kewei Li, Wenpeng Gu, Junrong Liang, Yuchun Xiao, Haiyan Qiu, Haoshu Yang, Xin Wang, Huaiqi Jing

**Affiliations:** National Institute for Communicable Disease Control and Prevention, Chinese Center for Disease Control and Prevention, State Key Laboratory for Infectious Disease Prevention and Control, Collaborative Innovation Center for Diagnosis and Treatment of Infectious Diseases, Beijing, 102206 China; Yunnan Provincial Centre for Disease Control and Prevention, 650022 Kunming, China

**Keywords:** *Yersinia enterocolitica*, *ompA*, *p*-*ompA*, *ystB*

## Abstract

**Background:**

*Yersinia enterocolitica* outer membrane protein A (OmpA) is one of the major outer membrane proteins with high immunogenicity. We performed the polymorphism analysis for the outer membrane protein A and putative outer membrane protein A (*p*-*ompA*) family protein gene of 318 *Y. enterocolitica* strains.

**Results:**

The data showed all the pathogenic strains and biotype 1A strains harboring *ystB* gene carried both *ompA* and *p*-*ompA* genes; parts of the biotype 1A strains not harboring *ystB* gene carried either *ompA* or *p*-*ompA* gene. In non-pathogenic strains (biotype 1A), distribution of the two genes and *ystB* were highly correlated, showing genetic polymorphism. The pathogenic and non-pathogenic, highly and weakly pathogenic strains were divided into different groups based on sequence analysis of two genes. Although the variations of the sequences, the translated proteins and predicted secondary or tertiary structures of OmpA and P-OmpA were similar.

**Conclusions:**

*OmpA* and *p*-*ompA* gene were highly conserved for pathogenic *Y. enterocolitica*. The distributions of two genes were correlated with *ystB* for biotype 1A strains. The polymorphism analysis results of the two genes probably due to different bio-serotypes of the strains, and reflected the dissemination of different bio-serotype clones of *Y. enterocolitica*.

## Background

*Y. enterocolitica* is spread primarily through contaminated food or water [1] and causes a wide range of intestinal diseases, including enteritis, mesenteric lymphadenitis, and sepsis in some severe cases; and also cause some complications such as erythema nodosum and reactive arthritis [2]. Currently, *Y. enterocolitica* is divided into six biotypes (1A, 1B, and 2–5) and more than 50 serotypes [3]. The biotypes of *Y. enterocolitica* are divided into three groups according to the bacterial pathogenic properties: non-pathogenic biotype 1A, weakly pathogenic biotypes 2–5, and highly pathogenic biotype 1B [4]. At present, the virulent factors of *Y. enterocolitica* are mainly referred to type III secretion system (TTSS) encoded by 70-kb plasmid pYV [3, 5], Yersinia adhesin A (YadA), the virulence genes involved *inv* (invasion gene), *ail* (attachment and invasion locus gene), *yst* (*Yersinia* stable toxin gene), *myfA* (mucoid *Yersinia* factor gene) and the pathogenic island (HPI), etc. [6]. Biotype 1A strains are traditionally considered non-pathogenic, however in recent studies have confirmed that a portion of them can cause clinical symptoms similar to pathogenic strains [7].

Bacterial outer membrane proteins primarily contain outer membrane protein A, porin C and F, being the major immunogenic proteins, and widely present in Gram-negative Enterobacteriaceae [8]. Recently, multifunction of outer membrane protein A of intestinal bacteria has been demonstrated [9–12], but seldom referred to *Y. enterocolitica*. In our previous study [13], OmpA was the major immunogenic protein of both highly and weakly pathogenic *Y. enterocolitica* incubated at different temperatures. To further identify the characteristics of *ompA* for *Y. enterocolitica*, we sequenced and analyzed the polymorphism of *ompA* (and *p*-*ompA*) genes of *Y. enterocolitica*.

## Results

### Distribution of *ompA*and *p*-*ompA*genes

The *ompA* and *p*-*ompA* genes were detected in 318 *Y. enterocolitica* strains. The data showed 170 of all the pathogenic strains carried both *ompA* and *p*-*ompA*; 91 biotype 1A strains carried *ompA*, and 106 carried *p*-*ompA* (Table 1). The *ompA* and *p*-*ompA* genes were both existed for biotype 1A strains carried *ystB* gene. However, the isolates only had one of the two genes for biotype 1A strains without *ystB* gene. The distribution of the two genes was associated with *ystB*, especially for *ompA* (Tables 2 and 3). The correlation analysis (P < 0.05) showed correlation coefficient (r) was 0.67 between *ompA* and *ystB* and 0.58 between *p*-*ompA* and *ystB*.

**Table 1:** **Distribution of** ***ompA*** **and** ***p***-***ompA*** **gene in all strains**

	***ompA*** +		***ompA*** -		Total
	***p***-***ompA***+	***p***-***ompA***-	***p***-***ompA***+	***p***-***ompA***-	
Pathogenic strains	170	0	0	0	170
Biotype 1A strains *ystB*+	84	0	0	0	84
Biotype 1A strains *ystB*-	0	7	22	35	64
Total	254	7	22	35	318

+: positive; -: negative.

**Table 2:** **Distribution of** ***ompA*** **and** ***ystB*** **gene in biotype 1A strains**

***ompA***	***ystB***		Total
	+	-	
+	84	7	91
-	0	57	57
Total	84	64	148

+: positive; -: negative.

**Table 3:** **Distribution of** ***p***-***ompA*** **and** ***ystB*** **gene in biotype 1A strains**

***p***-***ompA***	***ystB***		Total
	+	-	
+	84	22	106
-	0	42	42
Total	84	64	148

+: positive; -: negative.

### Polymorphism analysis of the two genes

**Figure 1:**
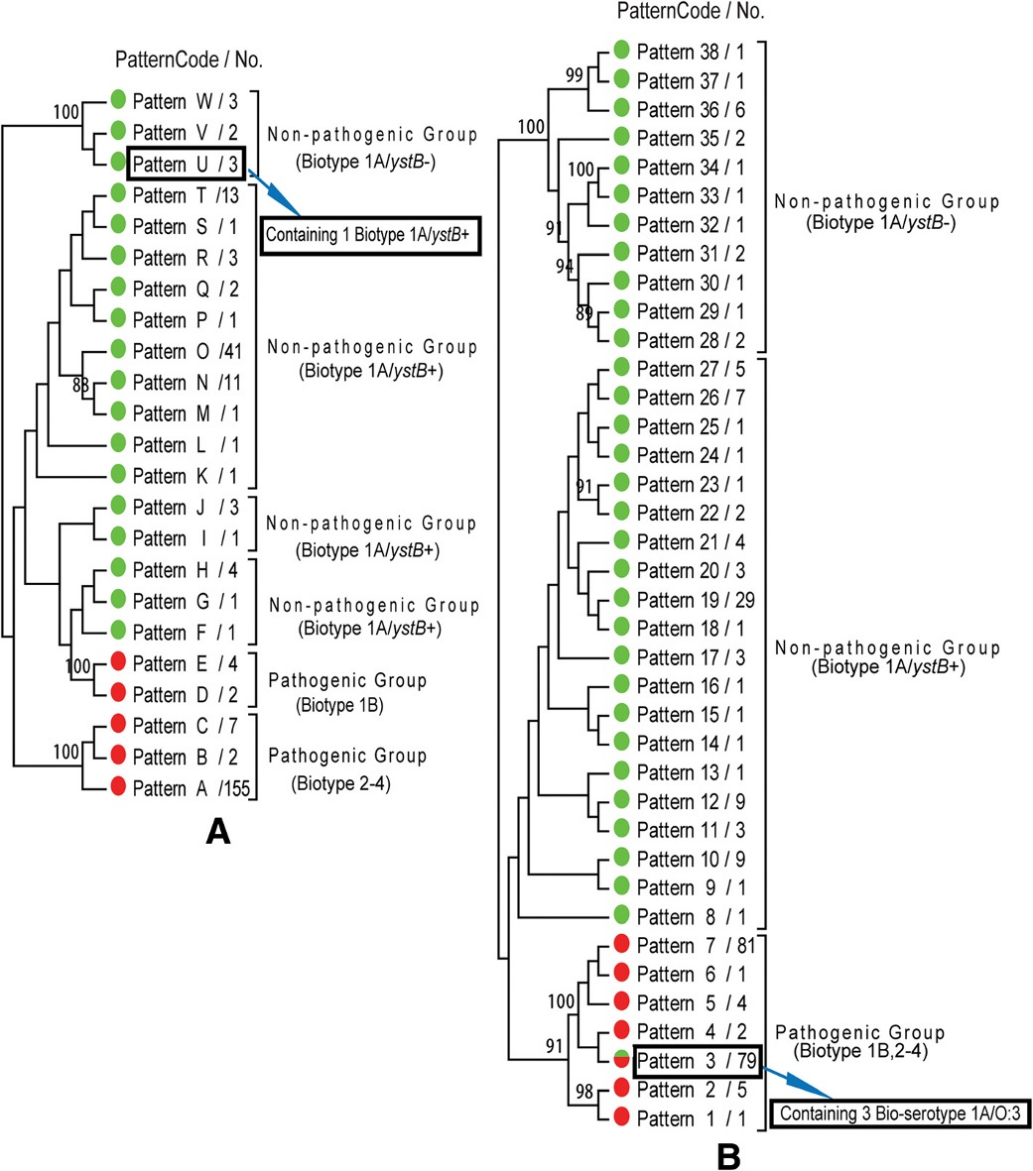
**Cluster tree of** ***ompA*** **and** ***p***-***ompA*** **gene sequences. A**: Cluster tree of *ompA* gene sequences from 261 strains; **B**: Cluster tree of *p*-*ompA* gene sequences from 275 strains; red: pathogenic strains; green: non-pathogenic strains.

**Figure 2:**
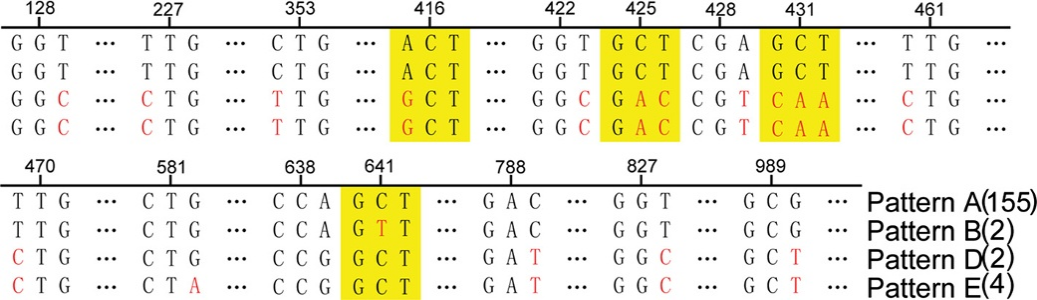
**Sequence polymorphisms of** ***ompA*** **gene for pathogenic strains.** The number above bases represented the position of bases in the ORF; figure in brackets represented strain number; red represented mutant bases; yellow area represented sense mutations, and others were nonsense mutations.

**Figure 3:**
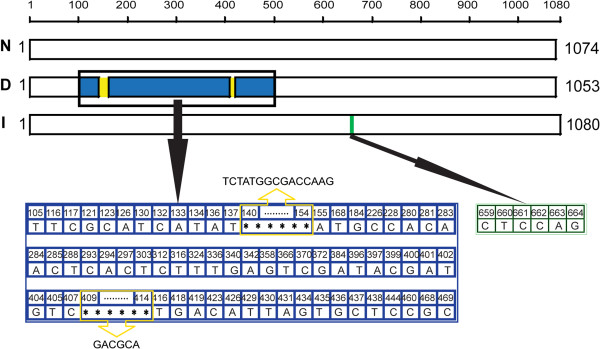
***ompA*** **nucleotide insertions and deletions.** N: the ORF of pattern A for *ompA*; D: the deletion ORF of pattern U-W for *ompA*; I: the insertion ORF of pattern C for *ompA*; blue: nucleotide differences concentrated area; yellow: nucleotide deletion position; green: nucleotide insertion position.

*ompA*: The Open Reading Frame (ORF) of *ompA* was 1,074 bp encoding 357 amino acids (Genbank: YP_001005874.1). The *ompA* gene of 261 strains formed 23 sequence types. The pathogenic group contained five types, Pattern A-E (Figure 1A). 155 pathogenic strains (76 bio-serotype 3/O: 3, two 4/O: 3, 68 2/O: 9, one 4/O: 9 and eight 3/O: 9) were clustered into Pattern A, and accounted for 91.2% of all of the pathogenic strains. Therefore, pattern A was an absolute primary type in all pathogenic isolates. Other nine pathogenic strains were clustered into pattern B and C, seven (two bio-serotype 2/O: 3, four 4/O: 3 and one 3/O: 9) were clustered into Pattern C, two pathogenic bio-serotype 2/O: 5, 27 clustered into pattern B. Few nucleotide differences were found between pattern A and B (Figure 2); while the nucleotide insertion was found compared with pattern A and C (Figure 3). Six highly pathogenic bio-serotype 1B/O: 8 strains were clustered into Patterns D and E (Figure 1A), and the nucleotide differences were shown with red bases compared with pattern A and B (Figure 2).

The non-pathogenic group contained 91 biotype 1A strains, formed 18 sequence types, Pattern F-W; all the 83 strains carried the *ystB* gene were clustered into pattern F-T; Eight strains (except one) without *ystB* gene formed pattern U-W (Figure 1A).

There were nucleotide fragment insertions and deletions for *ompA* gene sequences. The nucleotide fragment insertion was found for pattern C. The insertion site located at 659–664 nt of the ORF with the “-CTCCAG-” compared with pattern A (Figure 3I), this made an alanine (A) and a proline (P) inserted at position 220 and 221 in the amino acid sequence of the OmpA. For biotype 1A strains, sequence type diversities were found compared with pathogenic strains, and the nucleotide fragment deletions were observed for pattern U to W. 15 nucleotides deletions located at 140–154 nt with “-TCTATGGCGACCAAG-”, and 6 nucleotides deletions located at 409–414 nt with “-GACGCA-” of *ompA* were found (Figure 3D). Eventually, this led to a change of the amino acid of the OmpA at sites 39–52, 94–98,133-146, included amino acid deletions or translation into other amino acids.

Although some predicted amino acid differences were found for *ompA* of *Y. enterocolitica*, the primary proteins and its functions were the same, even if the insertions of the pathogenic or deletions of the non-pathogenic strains occurred. For all the *Y. enterocolitica* possessed *ompA* in this study, the translated proteins were identical. The predicted secondary structure of OmpA for all the strains were almost the same, and the predicted tertiary structure of OmpA for all the isolates were similar as well. It was highly conserved for the structure and function of OmpA for *Y. enterocolitica*, no matter the pathogenic ability and other characteristics.

**Figure 4:**
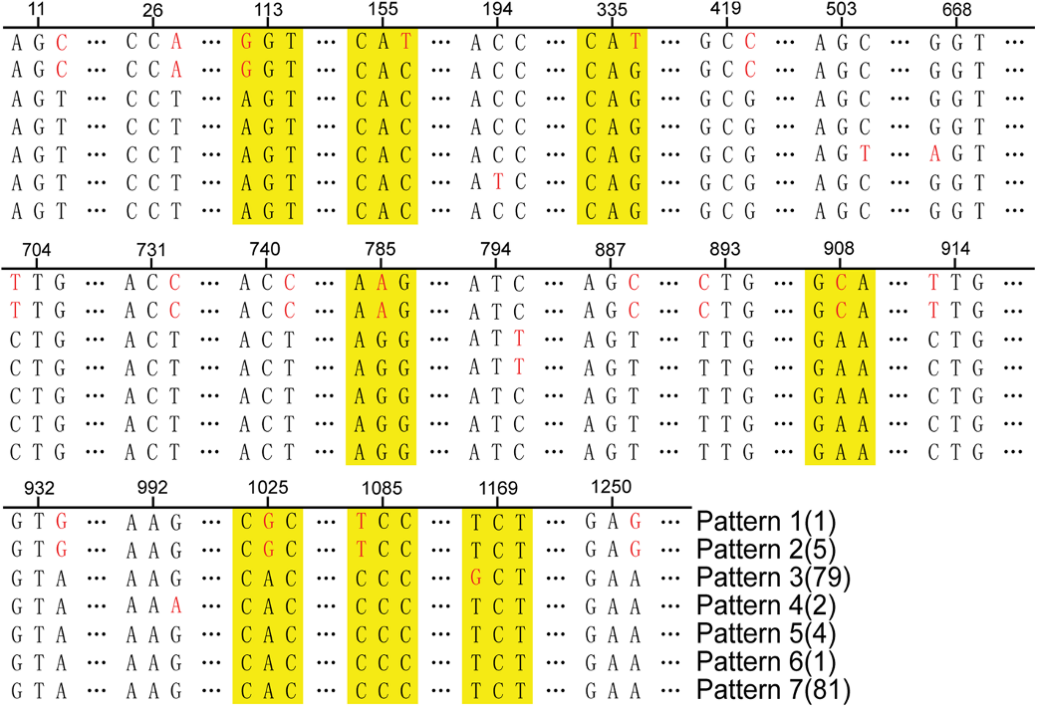
**Sequence polymorphisms of** ***p***-***ompA*** **gene for pathogenic strains.** The number above the bases represented position of the bases in the ORF; figure in brackets represents the strain number; red represented mutant bases; yellow area represented sense mutations, and others were nonsense mutations.

*p*-*ompA*: The Open Reading Frame (ORF) of *p*-*ompA* was 1,377 bp encoding 458 amino acids (GenBank: YP_001006877.1). *P*-*ompA* genes were existed among 276 *Y. enterocolitica*, 170 pathogenic and 106 biotype 1A strains (84 carried *ystB* gene and 22 without). The *p*-*ompA* genes were clustered into 38 sequence types, divided into pathogenic and non-pathogenic group (Figure 1B). Pattern 1–7 belonged to pathogenic group, included all the 170 pathogenic strains and three bio-serotype 1A/O: 3 isolates. Pattern 3 and pattern 7 were the primary types for the pathogenic strains, 67 bio-serotype 2/O: 9, eight 3/O: 9, one 4/O: 9 and three bio-serotype 1A/O: 3 strains formed pattern 3; 75 bio-serotype 3/O: 3, one 2/O: 3, four 4/O: 3 and one 3/O: 9 formed pattern 7. Pattern 5 contained two bio-serotype 4/O: 3, one 3/O: 3 and one 2/O: 9 strains; pattern 6 contained only one 2/O: 3 isolate; two bio-serotype 2/O: 5, 27 strains formed pattern 4, and six highly pathogenic 1B/O: 8 strains formed pattern 1 and 2. Some nucleotide point mutants were found among pattern 1 to 7, as shown in Figure 4.

Pattern 8–38 referred to non-pathogenic group, included 106 biotype 1A strains. All of the 84 isolates carried *ystB* were clustered into pattern 8 to 27; 22 strains without *ystB* were clustered into pattern 28 to 38 (Figure 1B).

Although the diversity of cluster results was found for *p*-*ompA*, the primary proteins structure and function of different patterns were identical predicted by software. The predicted secondary and tertiary structure or functions of proteins for all patterns were also similar, shown the conservative characteristic.

## Discussions

The surface structures of bacterial pathogens (including pilus, flagella, outer membrane proteins, and various secretion systems) are likely to interact with host tissue to regulate adhesion and invasion [14]. The outer membrane protein A belong to highly conserved protein in intestinal bacteria, and play a key role in bacterial integrity and virulence [15]. Currently, more evidence shows the pathogenicity of outer membrane protein A in a variety of pathogens [11, 16–19].

In our study, all the 170 pathogenic *Y. enterocolitica* strains carried the *ompA* and *p*-*ompA* genes. Few nucleotides changes were found for both *ompA* and *p*-*ompA* of the pathogenic strains. Therefore, the distributions of two genes were highly conserved for pathogenic *Y. enterocolitica*. The translated proteins or predicted structures of different patterns of the two genes were the same, certificated the conservative property of *ompA* and *p*-*ompA* for *Y. enterocolitica*. Several researches had been widely shown the distribution of outer membrane protein A among entero-pathogenic bacteria, and its important role in bacterial infection and immunogenicity [16–19]. However, seldom study referred to *ompA* or *p*-*ompA* of *Y. enterocolitica*, so it was the first time to perform this research. Our results showed the pathogenic strains and non-pathogenic strains were divided into different groups, and highly or weakly pathogenic strains were also distinguished based on sequence results of two genes, which reflected the different bio-serotype distributions of *Y. enterocolitica*. In China, serotype O: 3 and O: 9 strains were predominate pathogenic *Y. enterocolitica*, and most of these bacteria isolated from patients, swine and dogs. While, non-pathogenic strains referred to biotype 1A, and widely distributed among different hosts. Furthermore, no highly pathogenic 1B/O: 8 strain was isolated in China up to present, all the highly pathogenic 1B/O: 8 were foreign strains in our study. Additionally, the weakly pathogenic strains (biotype 2–4, serotype O: 3 or O: 9 strains) included wild strains from China and reference strains not from China showed no differences even if they were isolated from different origins in our study. Therefore, the cluster results for *ompA* or *p*-*ompA* explained the different bio-serotype distributions of *Y. enterocolitica*. Highly pathogenic biotype 1B strains have been shown to differ genetically from weakly pathogenic biotype 2–4 strains, and they belonged to different subtypes. The polymorphism analysis results of the two genes probably reflected the dissemination of different bio-serotype clones of *Y. enterocolitica* for a period of time.

Biotype 1A strains lack pYV plasmid and chromosomal virulence genes, and generally regarded as avirulent [1]. However, few studies have confirmed biotype 1A strains were related to outbreaks of nosocomial infections and foodborne diarrhea [20, 21]; and some early studies found that biotype 1A strains could cause abortion in goats and cattle [22–25]. Grant et al. [26, 27] showed biotype 1A strains invaded epithelial cells and resisted the killing effect of macrophage. Also biotype 1A strains were associated with the potential pathogenicity in humans [28]. Enterotoxin is an important pathogenic factor in most enteric pathogens, and the *ystB* gene coded for a class of thermo-resistant enterotoxin in biotype 1A *Y. enterocolitica*[29, 30]. Virulence related gene *ystB* was a distinguishing marker of biotype 1A strains, presented in close to 100% of clinical isolates [31, 32]. However, whether *ystB* gene as a virulent factor for biotype 1A of *Y. enterocolitica* has not been confirmed. Nakano et al. [33] found Salmonella enterotoxin (stn) regulated the OmpA membrane localization and functions, indicated the close relationship between them. In our research, *ompA* and *p*-*ompA* were correlated with *ystB* in biotype 1A strains, and formed the independent cluster patterns, the strains with *ystB* or without *ystB* for biotype 1A were also separated, which indicated the phenomenon that OmpA was linked with enterotoxins for *Y. enterocolitica* biotype 1A strains.

## Conclusions

We showed the *ompA* and *p*-*ompA* genes of *Y. enterocolitica* were highly conserved in pathogenic strains; specially, the two genes showed a high correlation with *ystB* in biotype 1A strains. The pathogenic and non-pathogenic strains, highly and weakly pathogenic strains were divided into different groups based on sequence polymorphism analysis of the two genes, which reflected the different bio-serotype distributions of *Y. enterocolitica*.

## Methods

### Bacterial strains and identification of biotype and serotype

The bacterial strains used in this study were screened from the Chinese Y*ersinia enterocolitica* library which contains nearly 4,000 strains gathered by our laboratory and derived from diarrhea patients, food, animals, and the environment. Strains were selected to cover different isolation dates, different hosts, and separated locations. We chose 150 pathogenic and 148 biotype 1A *Y. enterocolitica* strains isolated from China; 16 pathogenic reference strains from Europe, United States, and Japan; and four pathogenic complete-genome-sequenced strains (Table 4). The serotypes of these strains were determined as previously described [1, 34–36], and the biotypes of strains were identified using the scheme reviewed by Bottone [37]. The pathogenic strains were positive for all genes (*ail*^+^, *ystA*^+^, *virF*^+^, and *yadA*^+^); however, some pathogenic strains lost the plasmid virulence genes for *virF* and *yadA*, but still had *ail* and *ystA* genes located on the chromosome, the non-pathogenic strain was negative for all these genes.

**Table 4:** **The information of** ***Y. enterocolitica*** **used in this study**

Source	Pathogenic strains (bio-serotype)									Non-pathogenic strains (bio-serotype)						
	2/ O: 9	3/ O: 9	4/ O: 9	2/ O: 3	3/ O: 3	4/ O: 3	2/ O: 5, 27	1B/ O: 8	Total	1A/ O: 3	1A/ O: 8	1A/ O: 9	1A/ O; 5	1A/ O: 5, 27	1A/ UN	Total
Diarrhea patients	7	4			14	2			27		1	1	2		4	8
Swine	34	3			51				88		18	4	4		39	65
Dogs	2			1	5				8		2				7	9
Rats	19								19		2		2	2	1	7
Sheep				1					1		2	2		2	6	12
Cows										3	10	2			4	19
Fish					1				1							
Chickens	1	1							2		5	2	2		6	15
Ducks													1		2	3
Sparrows											1					1
Flies											1				1	2
Food	3								3		3	1			3	7
Environment	1								1							
Reference strains			1		5	3	2	5	16							
Sequence strains	1^a^	1^b^				1^c^		1^d^	4							
Total	68	9	1	2	76	6	2	6	170	3	45	12	11	4	73	148

a: *Y. enterocolitica* W22703, contig 7180000001374, GenBank: FR718562.1; b: *Y. enterocolitica* subsp. palearctica 105.5R (r), complete genome, GenBank: CP002246.1; c: *Y. enterocolitica* subsp. palearctica Y11, GenBank: FR729477.2; d: *Y. enterocolitica* subsp. enterocolitica 8081 complete genome, GenBank: AM286415.1; UN: undetermined serotype.

The sample collection and detection protocols were approved by the Ethics Review Committee from the National Institute for Communicable Disease Control and Prevention, Chinese Center for Disease Control and Prevention.

### Primer design

Two genes for OmpA of *Y. enterocolitica* reference strain 8081 (NC_008800.1) were shown when we searched the NCBI web, one was *ompA*, another was *p*-*ompA*. Therefore, we designed the primers of the two genes by using CloneManager software 4.0, and the primers were showed in Table 5. Primers were synthesized by Shanghai Sangon Biological Engineering & Technology and Service Co., Ltd, China.

**Table 5:** **Primers and annealing temperatures for** ***ompA*** **and** ***p***-***ompA***

Target gene	Primer direction	Primer Sequences (5′ → 3′)	GenBank no.	Location	Amplicon lengt	Annealing temp
*ompA*	Forward	ACATCACACTTGTAACTTTCTCACC	YP_001005874.1	1783285-1783261	1451 bp	58°C
	Reverse	AGAAGTATCAGAATCAGATGTCGTC		1781835-1781859		
*p*-*ompA*	Forward	GCGGCAAATTCCGTACAGTG	YP_001006877.1	2919405-2919386	1560 bp	60°C
	Reverse	CAGCCCACCAGCAATATTCG		2917806-2917825		

### PCR, DNA sequencing and sequence analysis

Bacteria were cultured as previously described [35]. The bacterial DNA was extracted using a Blood & Tissue Kit (QIAGEN, USA). PCR was performed in a 20 μl volume containing 10 μl PCR premix (TaKaRa, Japan), 8 μl ultrapure water, 0.5 μl of each forward and reverse primer (25 μmol/l), and 10 ng DNA template. Thermal cycling was performed in a MJ PTC200 (Bio-Rad, USA) and the conditions were: denaturation at 94°C for 5 min, followed by 25 cycles of melting at 94°C for 25 s, annealing for 30 s at various temperatures depending on the primers used (Table 5), elongation at 72°C for 30 s, and a final extension at 72°C for 10 min. The specific PCR products were purified using a Gel Extraction Kit (QIAGEN, USA) and sequenced at TaKaRa Biotechnology (Dalian) Co., Ltd. Nucleotide sequence alignments and cluster tree construction were performed using MEGA (Version 4). The statistical tests were performed using statistical analysis software SAS version 9.2 (Statistics Analysis System).

The different sequences of two genes were translated to predict amino acid by MEGA 4.0 software, and the second structure of the proteins were predicted by PredictProtein (http://www.predictprotein.org); the tertiary structure of proteins were predicted and analyzed by SWISS-MODEL (http://swissmodel.expasy.org/workspace).

## Availability of supporting data

All types of patterns for *ompA* and *p*-*ompA* of *Yersinia enterocolitica* in our study were uploaded to LabArchives (http://www.labarchives.com/bmc) Electronic Laboratory Notebook. All sequences can be shared from the following links:

*ompA* sequences:

https://mynotebook.labarchives.com/share/hqjing/MjAuOHwzMjk5OC8xNi0yL1RyZWVOb2RlLzMwNjA0MzQxNDh8NTIuOA=DOI:10.6070/H4MP517C.

*p*-*ompA* sequences:

https://mynotebook.labarchives.com/share/hqjing/MjIuMXwzMjk5OC8xNy00L1RyZWVOb2RlLzQwMDM0NDM2MTB8NTYuMQ= DOI:10.6070/H4GX48HN.
